# Association of cesarean birth with prevalence of functional constipation in toddlers at 3 years of age: results from the Japan Environment and Children’s Study (JECS)

**DOI:** 10.1186/s12887-021-02885-9

**Published:** 2021-09-23

**Authors:** Mari Nakamura, Kenta Matsumura, Yoshiko Ohnuma, Taketoshi Yoshida, Akiko Tsuchida, Kei Hamazaki, Hidekuni Inadera, the Japan Environment and Children’s Study Group, Michihiro Kamijima, Shin Yamazaki, Yukihiro Ohya, Reiko Kishi, Nobuo Yaegashi, Koichi Hashimoto, Chisato Mori, Shuichi Ito, Zentaro Yamagata, Hidekuni Inadera, Takeo Nakayama, Hiroyasu Iso, Masayuki Shima, Youichi Kurozawa, Narufumi Suganuma, Koichi Kusuhara, Takahiko Katoh

**Affiliations:** 1 Department of Public Health, Faculty of Medicine, University of Toyama, 2630 Sugitani, Toyama City, Toyama 930-0194 Japan; 2 Toyama Regional Center for JECS, University of Toyama, 2630 Sugitani, Toyama City, Toyama 930-8555 Japan; 3 Division of Neonatology, Maternal and Perinatal Center, Toyama University Hospital, 2630 Sugitani, Toyama City, Toyama 930-0194 Japan; 4 Department of Public Health, Gunma University Graduate School of Medicine, 3-39-22 Showa, Maebashi City, Gunma 371-8511 Japan

**Keywords:** Birth cohort, Cesarean section, Delivery mode, Functional constipation

## Abstract

**Background:**

The association between delivery mode and subsequent development of diseases is a growing area of research. Cesarean delivery affects the diversity of the microbiota in the infant gut, which may be associated with gastrointestinal disorders, including functional constipation, in infants. In this study, we investigated the association between delivery mode and prevalence of functional constipation in 3-year-old Japanese toddlers.

**Methods:**

This study used data from the Japan Environment and Children’s Study, an ongoing nationwide birth cohort study. We analyzed 71,878 toddler–mother pairs. The presence of functional constipation was determined according to the Rome III diagnostic criteria. Odds ratios and 95% confidence intervals were calculated using logistic regression analysis.

**Results:**

The prevalence of functional constipation in 3-year-old Japanese toddlers was estimated to be 12.3%. Logistic regression analysis revealed that the prevalence of functional constipation was higher in toddlers born by cesarean delivery (13.1%) compared with those born by vaginal delivery (12.1%), independent of 22 confounders (adjusted odds ratios = 1.064, 95% confidence interval = 1.004–1.128).

**Conclusions:**

We determined the prevalence of functional constipation in 3-year-old Japanese toddlers and found that delivery mode was associated with the prevalence of functional constipation in Japanese toddlers.

## Background

The association between delivery mode and the development of diseases later in childhood is a growing area of research. It has been suggested that Cesarean delivery (CD) may be associated with various diseases in infants, such as asthma, type 1 diabetes, inflammatory bowel disease, celiac disease, and obesity in later life [1–7]. A possible explanation for this is that CD differs from vaginal delivery (VD) in terms of verticalization, the process by which microbes are passed from the mother to her infant. During and after passage through the birth canal, VD exposes the neonate to a wide variety of microbes from the vaginal flora and intestinal microbiota. Infants born by CD, on the other hand, are not exposed to these same microbial communities and their microbiota more closely resembles the mother’s skin flora [8]. Consequently, CD might affect the microbiota diversity in the infant gut, which may lead to diseases later in life due to potential immune system impairment [2, 9, 10]. Children born by VD have more diverse intestinal microbiota compared with children born by CD [11]. In mice, initial exposure to microbes appears to have an effect on the gut microbiota and priming of the regulatory immune system [10]. Thus, the mode of delivery may be an important factor associated with the prevalence of disease later in life.

Among children with constipation, more than 90% are classified as having functional constipation (FC) because no organic cause can be found. FC has a significant impact on physical and emotional growth, which can lead to decreased quality of life as well as considerable healthcare costs [12, 13]. Investigating risk factors related to FC in early childhood may provide important clues for its etiology and help guide the development of preventive strategies. Abnormal intestinal flora may cause functional gastrointestinal disorders, including constipation, gastroesophageal reflux, and infantile colic [14]. Disruption of the intestinal microbiota is regarded as an etiological factor in pediatric FC [15]. In fact, a previous study has demonstrated that the colonic mucosal microbiota profile can be used to discriminate between healthy individuals and patients with constipation [16]. Therefore, it is possible that the prevalence of FC is greater in infants born by CD than in those born by VD.

The aim of this study was to examine the association between delivery mode and the development of FC at 3 years of age, based on data from a nationwide longitudinal study, the Japan Environment and Children’s Study (JECS). We furthermore aimed to reveal the prevalence of FC in Japanese 3-year-old toddlers.

## Methods

### Study design and participants

**Fig. 1:**
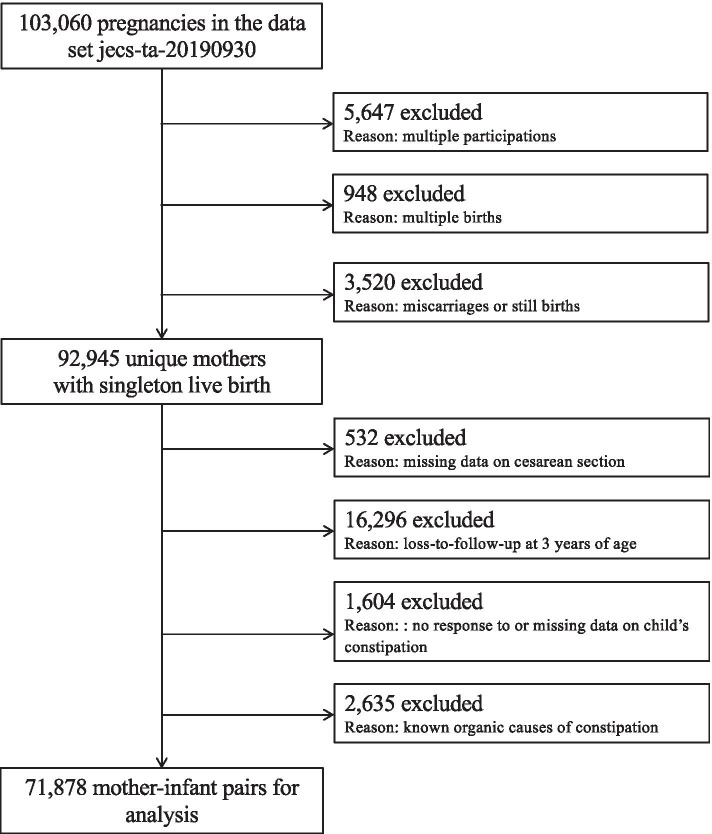
Participant flow diagram

The design of the JECS has been reported previously [17, 18]. Briefly, the JECS is a nationwide government-funded birth cohort study designed to investigate the effect of environmental factors on the health and development of children in Japan. A total of 103,060 pregnancies were registered at 15 regional centers from Hokkaido in the north to Okinawa in the south between January 2011 and March 2014. The present study analyzed the *jecs-ta-20190930* data set (released October 2019), which includes prospectively collected data on toddlers up to 3 years of age as well as their mothers. We excluded 5,647 multiple participations (i.e., the second or third registration of the same mother), 948 multiple births (e.g., twins or triplets), 3,520 miscarriages/stillbirths, 532 missing data on cesarean section, 16,296 lost to follow-up at 3 years of age, and 1,604 insufficient or missing data on the child’s constipation. Participants with known organic causes of constipation, including Hirschsprung’s disease, spina bifida, thyroid gland insufficiency, and allergy to cow’s milk that were diagnosed by physicians and reported by mothers, were also excluded, leaving 71,878 mother–toddler pairs for the final analysis (Fig. 1).

### Measures

#### Exposure

Data on CD was derived from medical record transcriptions performed by physicians, midwives/nurses, and/or research coordinators.

#### Outcome

Toddlers at 3 years of age were considered to have FC when their mother’s answers satisfied the Rome III diagnostic criteria for FC [19]. Rome III is a standard set of diagnostic criteria for childhood functional gastrointestinal disorders and requires two or more of the following items for a minimum of 1 month for a diagnosis of FC: 1. two or fewer defecations per week; 2. at least 1 episode per week of incontinence after the acquisition of toileting skills; 3. history of excessive stool retention, 4. history of painful or hard bowel movements; 5. presence of a large fecal mass in the rectum; 6. history of large-diameter stools that may obstruct the toilet. The Japanese version of Rome III was used in this study [20].

#### Statistical analysis

The outcome was cases of FC defined as any toddler satisfying the Rome III diagnostic criteria. The exposure variable was CD. Crude and adjusted odds ratios (CORs and AORs) as well as the corresponding 95% confidence intervals (CIs) were calculated by logistic regression analysis. Covariates were entered into the multivariable analysis model by the forced entry method. The JECS prohibits sharing the ORs of covariates, regardless of whether they are crude or adjusted. Missing data were also included in the model as dummy coded variables. Statistical analysis was carried out using SAS (version 9.4; SAS Institute Inc., Cary, NC).

### Covariates

Based on our previous study investigating constipation at age 1 year [21], we included the following covariates for children in the analysis: infant sex (male or female), major congenital anomaly (yes or no) [22], gestational age (<34, 34–36, 37–40, >41 weeks), started solid food (including fruit juice or rice gruel) at 6 months (yes or no), breastfeeding (the child has already been weaned or was formula-fed, yes at least till 1 year, yes at least till 2 years), attendance at a childcare facility (daycare center/nursery) at 1 year (yes or no), attendance at a preschool or childcare facility (daycare center/nursery) at 3 years (yes or no), constipation at 1 year (yes or no) [23]. As covariates for mothers, we included age during pregnancy (<25, 25 to <30, 30 to <35, ≥35 years), pre-pregnant body mass index (<18.5, 18.5 to <25, ≥25), parity (primipara or multipara), maternal history of allergy (yes or no), use of antibacterial drug during pregnancy (yes or no), annual household income (<4, 4 to <6, ≥6 million JPY), highest education level (≤12, >12 to <16, ≥16 years), employed during pregnancy (yes or no), marital status (married, single, divorced or widowed), physical activity corresponding to 10 min of walking (yes or no) [24, 25], quintile of energy intake (Q1 [≤1,228], Q2 [1,229–1,490], Q3 [1,491–1,750], Q4 [1,751–2,127], Q5 [≥2,128], cal) measured using a food-frequency questionnaire [26], smoking status at 1 month postpartum (previously smoked but quit before finding out about the current pregnancy, previously smoked but quit after finding out about the current pregnancy, still smokes [1–10 per day], still smokes [≥11 per day]), passive smoking status at 1 month (no one smoked; someone smoked but not in the presence of the baby, somebody smoked in the presence of the baby), and alcohol consumption at 1 month (not a drinker, drank in the past but stopped drinking, still drinks). The variables were categorized according to standard medical practice, common practice in Japan, and/or based on previous studies [27].

## Results

The 71,878 mother–infant pairs were analyzed. In the toddler population, 18.6% were delivered by CS, 51.0% were male, 41.6% were exclusively breastfed at 1 month, and 1.3% had constipation at 1 year of age (Table 1). In the mother population, 72.5% were below 35 years of age, 73.8% had a pre-pregnancy body mass index of 18.5 to <25, and 43.0% were primipara (Table 1). The frequency of CS (20.1%) in those who were excluded (n = 21,067) from the study was higher than that in those (18.6%) who were included (n = 71,878).

**Table 1:** Characteristics of the participants (n = 71,878 mother–toddler pairs)

	Cesarean section				
	No		Yes		
	(n = 58,530)		(n = 13,348)		
	n	(%)	n	(%)	*p*
Toddler					
Infant sex					
Male	29,855	(51.0)	6,809	(51.0)	0.994
Female	28,675	(49.0)	6,539	(49.0)	
Any major congenital anomaly					
No	57,394	(98.1)	12,892	(96.6)	< 0.001
Yes	1,136	(1.9)	456	(3.4)	
Gestational age, wk					
≥41	5,786	(9.9)	1,012	(7.6)	< 0.001
37–40	50,898	(87.0)	10,914	(81.8)	
34–36	1,654	(2.8)	965	(7.2)	
<34	192	(0.3)	457	(3.4)	
Started solid food at 6 months					
No	42,934	(73.4)	9,367	(70.2)	< 0.001
Yes	14,669	(25.1)	3,744	(28.1)	
Missing	927	(1.6)	237	(1.8)	
Breastfeeding					
Already weaned or formula-fed	21,989	(37.6)	5,701	(42.7)	< 0.001
Yes at least till 1 year	25,749	(44.0)	5,156	(38.6)	
Yes at least till 2 years	9,546	(16.3)	2,226	(16.7)	
Missing	1,246	(2.1)	265	(2.0)	
Attendance at a childcare facility (daycare center/nursery) at 1 year					
No	42,262	(72.2)	9,661	(72.4)	0.541
Yes	14,763	(25.2)	3,366	(25.2)	
Missing	1,505	(2.6)	321	(2.4)	
Attendance at a preschool or childcare facility (daycare center/nursery) at 3 years					
No	21,218	(36.3)	4,658	(34.9)	0.012
Yes	35,579	(60.8)	8,278	(62.0)	
Missing	1,733	(3.0)	412	(3.1)	
Constipation at 1 year					
No	56,204	(96.0)	12,855	(96.3)	0.321
Yes	776	(1.3)	164	(1.2)	
Missing	1,550	(2.7)	329	(2.5)	
Mother					
Age during pregnancy, y					
<25	5,705	(9.8)	802	(6.0)	< 0.001
25 to <30	17,245	(29.5)	2,842	(21.3)	
30 to <35	20,950	(35.8)	4,635	(34.7)	
≥35	14,286	(24.4)	4,960	(37.2)	
Missing	344	(0.6)	109	(0.8)	
Pre-pregnancy body mass index, kg/m^2^					
<18.5	9,947	(17.0)	1,719	(12.9)	< 0.001
18.5 to <25	43,514	(74.3)	9,523	(71.3)	
≥25	5,036	(8.6)	2,096	(15.7)	
Missing	33	(0.1)	10	(0.1)	
Parity					
Primipara	25,128	(42.9)	5,759	(43.2)	0.902
Multipara	31,904	(54.5)	7,250	(54.3)	
Missing	1,498	(2.6)	339	(2.5)	
Maternal history of allergy					
No	29,208	(49.9)	6,663	(49.9)	0.993
Yes	28,989	(49.5)	6,608	(49.5)	
Missing	333	(0.6)	77	(0.6)	
Use of antibacterial drug during pregnancy					
No	52,370	(89.5)	11,906	(89.2)	0.345
Yes	6,160	(10.5)	1,442	(10.8)	
Annual household income, million Japanese yen					
<4	21,211	(36.2)	4,783	(35.8)	0.047
4 to <6	18,282	(31.2)	4,098	(30.7)	
≥6	14,882	(25.4)	3,549	(26.6)	
Missing	4,155	(7.1)	918	(6.9)	
Highest education level, y					
≤12	19,602	(33.5)	4,600	(34.5)	< 0.001
>12 to <16	24,733	(42.3)	5,737	(43.0)	
≥16	13,578	(23.2)	2,836	(21.3)	
Missing	617	(1.1)	175	(1.3)	
Employed during pregnancy					
No	26,177	(44.7)	5,982	(44.8)	0.025
Yes	31,553	(53.9)	7,143	(53.5)	
Missing	800	(1.4)	223	(1.7)	
Marital status					
Married	55,577	(95.0)	12,728	(95.4)	< 0.001
Single	2,004	(3.4)	383	(2.9)	
Divorced or widowed	386	(0.7)	118	(0.9)	
Missing	563	(1.0)	119	(0.9)	
Physical activity					
No	13,625	(23.3)	3,404	(25.5)	< 0.001
Yes	44,424	(75.9)	9,802	(73.4)	
Missing	481	(0.8)	142	(1.1)	
Quintile of energy intake, cal					
Q1 (≤1,228)	11,389	(19.5)	2,566	(19.2)	0.001
Q2 ( 1,229–1,490)	11,934	(20.4)	2,583	(19.4)	
Q3 ( 1,491–1,750)	11,807	(20.2)	2,697	(20.2)	
Q4 ( 1,751–2,127)	11,687	(20.0)	2,680	(20.1)	
Q5 (≥2,128)	11,368	(19.4)	2,712	(20.3)	
Missing	345	(0.6)	110	(0.8)	
Smoking status at 1 month					
Never smoked	35,428	(60.5)	7,755	(58.1)	< 0.001
Previously smoked but quit before finding out about the current pregnancy	12,936	(22.1)	3,126	(23.4)	
Previously smoked but quit after finding out about the current pregnancy	7,795	(13.3)	1,815	(13.6)	
Still smokes (1–10 per day)	1,558	(2.7)	394	(3.0)	
Still smokes (≥11 per day)	368	(0.6)	95	(0.7)	
Missing	445	(0.8)	163	(1.2)	
Passive smoking status at 1 month					
No one smoked	28,634	(48.9)	6,564	(49.2)	< 0.001
Someone smoked but not in the presence of the baby	28,212	(48.2)	6,308	(47.3)	
Someone smoked in the presence of the baby	1,295	(2.2)	298	(2.2)	
Missing	389	(0.7)	178	(1.3)	
Alcohol consumption at 1 month					
Never drank	53,390	(91.2)	12,045	(90.2)	< 0.001
Drank in the past but stopped drinking	2,535	(4.3)	603	(4.5)	
Still drinks	2,205	(3.8)	538	(4.0)	
Missing	400	(0.7)	162	(1.2)	

The answers to Rome III items are summarized in Table 2. Among the 6 items, history of painful or hard bowel movements was most prevalent (20.5%). The yes-or-no responses to the 6 items allow for 2^6^ (=64) different possible patterns of answers, of which 55 satisfied the diagnostic criteria and included at least 2 of the 6 items for a minimum of 1 month. The top 5 most common combinations were items 4 and 5 (n = 1,535; 17.4%); 3 and 4 (n = 1,322; 15.0%); 3, 4, and 5 (n = 717; 8.1%); 2 and 4 (n = 683; 7.8%); and 1 and 4 (n = 487; 5.5%).

**Table.2:** Answers to each Rome III item (n = 71,878 mother–toddler pairs)

	“Yes” answer	
	n	(%)^a^
1. Two or fewer defecations per week	3,315	(4.6)
2. At least 1 episode per week of incontinence after the acquisition of toileting skills	4,609	(6.4)
3. History of excessive stool retention	5,795	(8.1)
4. History of painful or hard bowel movements	14,704	(20.5)
5. Presence of a large fecal mass in the rectum	4,787	(6.7)
6. History of large-diameter stools that may obstruct the toilet	2,040	(2.8)

^a^All the denominators were 71,878

Overall, 12.3% of the toddlers (n = 8,811) had FC at 3 years of age. Logistic regression analysis revealed that the prevalence of FC was higher in toddlers born by CD (13.1%) compared with those born by VD (12.1%), independent of 22 confounders (AOR = 1.064, 95% CI = 1.004–1.128). These results are summarized in Table 3.

**Table.3:** Odds ratios [95% CIs] for cases of functional constipation at 3 years of age according to cesarean section (n = 71,878 mother–toddler pairs)

	Cesarean section	
	No	Yes
Prevalence, %	12.1	13.1
Cases, n	7,060	1,751
Subtotal, n	58,530	13,348
Crude odds ratio	1.000 (Ref.)	**1.101 [1.041, 1.164]**
Adjusted odds ratio^a^	1.000 (Ref.)	**1.064 [1.004, 1.128]**

Boldface indicates statistical significance at the level of two-sided *p* values of <5%

^a^Adjusted for infant sex, major congenital anomaly, gestational age, solid food at 6 months, breastfeeding, attending a childcare facility at 1 year, attending a preschool or childcare facility at 3 years, constipation at 1 year, maternal age during pregnancy, pre-pregnant body mass index, parity, maternal history of allergies, use of antibacterial drugs during pregnancy, annual household income, highest education level, employed during pregnancy, marital status, physical activity corresponding to 10 min of walking, quintile of energy intake, smoking status at 1 month postpartum, passive smoking status at 1 month, and alcohol consumption at 1 month

## Discussion

In this study, we revealed the prevalence of FC among 3-year-old toddlers in a large Japanese cohort. Moreover, we found that at 3 years of age, children born by CD have a significantly higher OR for FC compared with those born by VD. In a previous study, we analyzed the association between the mode of delivery and the development of constipation at age 1 year using the same cohort [21]. At 1 year of age, CD had no association with the frequency of bowel movements, although fecal incontinence in children wearing diapers is difficult to assess. Taken together, these results show that the prevalence of FC increased with age and that FC was reported at a significantly higher frequency in children born by CD (13.1%) at 3 years of age than in those born by VD (12.1%). Several previous studies also found that the prevalence of FC in childhood increased with age [28, 29], although the underlying reasons remain to be clarified.

FC has a significant impact on quality of life, including school absenteeism, and is considered to be a major public health issue among young children [30]. Given that so few patients with FC seek treatment, the exact prevalence is difficult to ascertain. A systematic review reported a mean prevalence of 14% in children [31]. A population-based study in the US found that 9.4% of toddlers had FC [28]. A study from Sri Lanka reported that 8% of infants and toddlers had FC [32]. The prevalence of FC was 8.5% in young children in Korea [33]. In the present study, the prevalence of FC in Japanese toddlers at age 3 years was estimated to be 12.3%. To our knowledge, this is the first study to report the prevalence of FC in a large cohort of Japanese toddlers. The differences in prevalence among countries might be attributable to differences in genetic, environmental, and social factors such as dietary patterns, and cultural differences related to toilet training and child rearing. In the future, it will be necessary to conduct studies at the international level, using the same methods and defined age groups in order to understand the global epidemiology of FC in young children.

Dietary intake, physical activity, psychological factors, and socioeconomic status may all play roles in the pathophysiology of FC. Previous studies have revealed that socioeconomic factors such as annual household income and mothers’ educational attainment were associated with lower prevalence of FC in children [31, 34]. A possible explanation relates to the different dietary habits and lifestyle associated with low socioeconomic status, which might affect the risk of developing FC. Our results showed that constipation at age 1 year had a higher AOR (4.40) for FC at age 3 years compared with other factors. Thus, gastrointestinal function during infancy may affect the development of FC at later stages of childhood.

It is well recognized that differences in the composition of gut microbiota in infants depend on delivery mode [35, 36]. The vertical transmission of maternal microbes to infants is a critical factor in the establishment of a core gut microbiota. Indeed, the microbiota of vaginally delivered neonates resembles the vaginal microbiota of their mother, while those of neonates born by CD resembled the mother’s skin microbiota [8, 37]. Infants born by CD have been shown to have low bacterial richness and diversity [11]. Thus, differences in mode of delivery have been linked to differences in the intestinal microbiota of infants. One study reported that the mode of delivery is associated with differences in intestinal microbes at 7 years of age [38]. Although the gut microbiota of healthy adults is thought to be stable over time, that of infants needs to develop before reaching maturity [39]. In developed countries, including Japan, rates of CD have continued to increase, and thus strategies for reducing the prevalence of FC, particularly in toddlers born by CD, will be necessary in the future.

A major strength of our study is its large sample size, which used the data of mother–toddler pairs obtained from 15 different regions of Japan. By conducting such a wide-ranging, large-scale study using prospectively collected data, unbiased results representative of the Japanese toddler population have been obtained [18], and the results should be extrapolatable to the Japanese general population. Furthermore, because the symptom-based questionnaire used to measure FC has been validated, its reliability is assured. Moreover, we were able to construct statistical models taking into account many independent variables, including maternal characteristics as well as socioeconomic and lifestyle factors. However, some limitations should be considered when interpreting the results. First, CD was not analyzed in terms of whether it was acute or elective because these data were not collected in the JECS. Compared with other children, children born by acute CD tend to have a lower Apgar score, and are at greater risk of ischemia, which can influence bowel motility as well as the gut microbiota. The inability to investigate differences between types of CD is thus considered a major limitation of this study. Second, 21,067 (22.7%) out of 92,945 mother−toddler pairs were excluded from the analysis. Among them, 16,296 were lost to follow-up and 1,604 were due to missing data on the child’s constipation, which may constitute a selection bias. Third, we used 22 covariates, but theoretically it is impossible to include all confounders. Thus, we cannot deny the possibility that the observed associations were spurious. Finally, the JECS relied on mothers to provide details of their children via a self-administered questionnaire. Information regarding medication and dietary factors that might influence gastrointestinal motility were not collected and thus may have biased our results.

## Conclusions

This study reported the prevalence of FC in Japanese toddlers at 3 years of age. FC was reported at a significantly higher frequency in children born by CD (13.1%) than in those born by VD (12.1%). Future studies are warranted to investigate ways to modify disturbed gut microbiota, such as administering probiotic supplementation, in order to reduce the occurrence of FC in early childhood.

## Data Availability

Data are unsuitable for public deposition due to ethical restrictions and the legal framework of Japan. It is prohibited by the Act on the Protection of Personal Information (Act No. 57 of 30 May 2003, amendment on 9 September 2015) to publicly deposit data containing personal information. Ethical Guidelines for Medical and Health Research Involving Human Subjects enforced by the Japan Ministry of Education, Culture, Sports, Science and Technology and the Ministry of Health, Labour and Welfare also restricts the open sharing of the epidemiologic data. All inquiries about access to data should be sent to: jecs-en@nies.go.jp. The person responsible for handling enquiries sent to this e-mail address is Dr Shoji F. Nakayama, JECS Programme Office, National Institute for Environmental Studies.

## Abbreviations

JECS: Japan Environment and Children’s Study
CD: Cesarean delivery
CS: Cesarean section
FC: Functional constipation
VD: Vaginal delivery
